# Hypocretin neuron hyperexcitability in the hypothalamus: a newly discovered culprit in aging-related sleep impairment

**DOI:** 10.1038/s41392-022-01091-y

**Published:** 2022-07-15

**Authors:** Tianbai Li, Congcong Jia, Weidong Le

**Affiliations:** 1 Liaoning Provincial Key Laboratory for Research on the Pathogenic Mechanisms of Neurological Diseases, the First Affiliated Hospital, Dalian Medical University, 116021 Dalian, China; 2 Institute of Neurology, Sichuan Academy of Medical Sciences, Sichuan Provincial Hospital, 610072 Chengdu, China

**Keywords:** Molecular neuroscience, Neurological disorders

The recent research published in Science by Li et al.^1^ provides compelling evidence that the hyperexcitability of arousal-promoting hypocretin (Hcrt, also called orexin) neurons is closely associated with sleep instability during aging (Fig. 1). Their findings reveal the underlying neural mechanisms of aging-related sleep impairment.

**Fig. 1:**
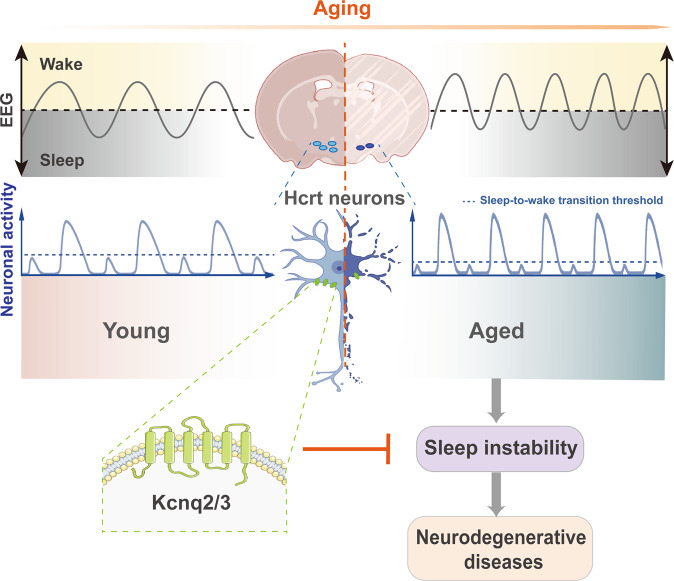
Role of the hyperexcitable Hcrt neurons in aging-related sleep instability and neurodegenerative diseases. Aged mice exhibit more frequency of sleep-wake transition, along with elevated excitability of Hcrt neurons compared with young mice. Kcnq2/3 channels in Hcrt neurons could be a promising therapeutic strategy to improve sleep continuity in the aged, thereby preventing neurodegenerative diseases

Sleep quality gradually declines with aging process due to reduced ability to initiate and maintain sleep. It has been shown that less consolidated sleep with more arousals or transitions to lighter sleep stages is common in the elderly. In a shorter term, aggravated sleep instability may lead to cognitive decline; in a longer term, it may cause neurodegeneration or fatal disorders.^2^ However, the neurobiological mechanisms contributing to aging-related sleep impairment are not fully understood. In recent years, extensive circuit-based investigations have been carried out from specific brain regions and neurons in the arousal regulatory systems to identify their functions in regulating sleep and wakefulness. Hcrt-producing neurons in the lateral hypothalamus are essential for sleep-wake regulation through complex interactions among monoaminergic/cholinergic (wake-promoting) and γ-aminobutyric acidergic (sleep-promoting) neuronal circuits.^3^ The electrophysiological study further demonstrates that the intrinsic excitability of Hcrt neurons is closely associated with sleep-wake transitions.^1,3^

Does the hyperexcitability of Hcrt neurons contribute to sleep instability during aging? Li et al. raised this question and linked Hcrt neuronal circuits to aging-related sleep impairment. In their study, aged mice exhibited more significant fragmented sleep correlated with a substantial loss (~38%) of Hcrt neurons in the hypothalamus as compared with young mice. Fiber photometry recording in aged mice showed that Hcrt neurons have more frequent firing patterns highly relevant to wake bouts and sleep fragmentation. Data from patch-clamp recording further verified the elevated intrinsic excitability of Hcrt neurons in aged mice with more depolarized membrane potentials (RMPs).^1^ Moreover, optogenetic stimulation of Hcrt neurons triggered a shorter latency time of sleep-to-wake transition in aged mice.^1^ These findings integrally characterize the electrophysiology properties of Hcrt neurons in the aging-related sleep instability. It is likely that altered membrane threshold and spontaneous firing activities of Hcrt neurons may contribute to the excitation-inhibition imbalances in Hcrt connectomics, thus disturbing the coordination of wake/sleep-promoting neuronal circuits. Li’s study emphasizes the necessity of repressing the hyperexcitability of aged Hcrt neurons to maintain sleep quality.

In addition, Li et al. explored the molecular mechanisms underlying the hyperexcitability in Hcrt neurons during aging.^1^ They discovered dysfunction of repolarizing M-current mediated by Kcnq2/3 channels and deficiency of Kcnq2 through array tomography at the ultrastructural resolution, and identified the dysregulated Kcnq family subtypes Kcnq1/2/3/5 by single-nucleus RNA-sequencing (snRNA-seq) in aged Hcrt neurons.^1^ Furthermore, they found that exerting CRISPR/SaCas9–mediated disruption of Kcnq2/3 genes in Hcrt neurons of young mice can mimic the aging-related sleep fragmentation features. Moreover, administration of Kcnq2/3-selective activator, flupirtine, significantly inhibited spontaneous firing activities in Hcrt neurons and increased the sleep stability in aged mice.^1^ These findings may provide a possible strategy to improve aging-related sleep disorders, and identifying more specific pharmacological targets acting on Hcrt neurons may shed light on conquering the possible unanticipated side effects of Hcrt receptor antagonists.

Hcrt neurons not only participate in regulating aging-related sleep impairment but also related to several other sleep disorders. Among them, the most noteworthy is narcolepsy, a sleep disorder at the early onset with sudden sleep disruption and cataplexy caused by a massive loss of Hcrt neurons or defects in Hcrt receptor 2. Li et al. pointed out that the basic electrophysiological properties and underlying mechanisms in narcolepsy mice are different from those in healthy aged mice,^1^ suggesting that the hyperexcitability in Hcrt neurons specifically emerges in aging. In addition, beyond normative aging, sleep disruption is especially pervasive in neurodegenerative diseases, such as Alzheimer’s disease.^4^ The activated pathway of Hcrt neurons-to-basal forebrain increases acetylcholine release and activity of cortical neurons, affecting cognitive function.^5^ Besides, the amyloid-β-induced hyperexcitation is identified to be correlated with the expression of Kcnq2/3 in hippocampal neurons.^5^ The hyperexcitability of Hcrt neurons in aged mice of this study may provide a theoretical basis for neurodegenerative diseases-associated sleep instability.

Sleep regulation involves a complex of neural pathways and networks in the brain. In addition to the Hcrt system, there are several other interacted sleep-wake regulation circuits, involving the noradrenergic, cholinergic, and dopaminergic systems. Furthermore, the circadian rhythm regulated by a group of clock genes also plays a vital role in controlling the sleep-wake cycle.^4^ It is currently unknown whether the circadian rhythm of Hcrt neurons is involved in aging-related sleep impairment, which deserves further investigation in the future. Besides, although this study has sufficient data to demonstrate the vital role of Hcrt neuronal activity in regulating sleep stability in aged mice, it still lacks evidence from human studies. Therefore, it is of importance to carry out the study on older adults who suffer from sleep disorders to verify whether there is an altered level of Kcnq2/3 in the Hcrt neuron. Furthermore, molecular imaging to probe the change of Hcrt neurons in humans may help clarify the correlations between the Hcrt neuronal activity and sleep stability.

Overall, Li et al. innovatively discovered an essential role of the arousal-promoting Hcrt system in regulating sleep during aging. Their findings indicate that the hyperexcitability of Hcrt neurons might be an important culprit contributing to the sleep instability in the aged. The pharmacological remedy of sleep disruption by targeting Kcnq2/3 channels could be a promising therapeutic strategy for improving sleep quality and preventing aging-related sleep disorders.

## References

[1] Li SB (2022). Hyperexcitable arousal circuits drive sleep instability during aging. Science.

[2] Zhang F (2019). Alteration in sleep architecture and electroencephalogram as an early sign of Alzheimer’s disease preceding the disease pathology and cognitive decline. Alzheimers Dement.

[3] Scammell TE, Arrigoni E, Lipton JO (2017). Neural circuitry of wakefulness and sleep. Neuron.

[4] Wang X (2022). Influence of sleep disruption on protein accumulation in neurodegenerative diseases. Ageing Neur Dis..

[5] Li SB (2020). The hypocretin (orexin) system: from a neural circuitry perspective. Neuropharmacology.

